# Stereotactic body radiation therapy with concurrent full-dose gemcitabine for locally advanced pancreatic cancer: a pilot trial demonstrating safety

**DOI:** 10.1186/1748-717X-8-44

**Published:** 2013-03-01

**Authors:** Marie K Gurka, Sean P Collins, Rebecca Slack, Gary Tse, Aline Charabaty, Lisa Ley, Liam Berzcel, Siyuan Lei, Simeng Suy, Nadim Haddad, Reena Jha, Colin D Johnson, Patrick Jackson, John L Marshall, Michael J Pishvaian

**Affiliations:** 1 Department of Radiation Oncology, Georgetown University Hospital, 20007, Washington, DC, USA; 2 Lombardi Comprehensive Cancer Center, Georgetown University, Podium B 3800 Reservoir Road, NW, 20007, Washington, DC, USA; 3 Department of Biostatistics, University of Texas MD Anderson Cancer Center, 77230, Houston, TX, USA; 4 Division of Gastroenterology, Department of Medicine, Georgetown University Hospital, 20007, Washington, DC, USA; 5 Department of Radiology, Georgetown University Hospital, 20007, Washington, DC, USA; 6 University Surgical Unit, Southampton University Hospitals, Southampton, United Kingdom; 7 Department of Surgery, Georgetown University Hospital, 20007, Washington, DC, USA

**Keywords:** Pancreatic cancer, Gemcitabine, Radiation, Stereotactic body radiation therapy, SBRT, CyberKnife, Quality of life, QLQ-C30, QLQ-PAN26, Upper endoscopy

## Abstract

**Background:**

Concurrent chemoradiation is a standard option for locally advanced pancreatic cancer (LAPC). Concurrent conventional radiation with full-dose gemcitabine has significant toxicity. Stereotactic body radiation therapy (SBRT) may provide the opportunity to administer radiation in a shorter time frame with similar efficacy and reduced toxicity. This Pilot study assessed the safety of concurrent full-dose gemcitabine with SBRT for LAPC.

**Methods:**

Patients received gemcitabine, 1000 mg/m^2^ for 6 cycles. During week 4 of cycle 1, patients received SBRT (25 Gy delivered in five consecutive daily fractions of 5 Gy prescribed to the 75-83% isodose line). Acute and late toxicities were assessed using NIH CTCAE v3. Tumor response was assessed by RECIST. Patients underwent an esophagogastroduodenoscopy at baseline, 2, and 6 months to assess the duodenal mucosa. Quality of life (QoL) data was collected before and after treatment using the QLQ-C30 and QLQ-PAN26 questionnaires.

**Results:**

Between September 2009 and February 2011, 11 patients enrolled with one withdrawal during radiation therapy. Patients had grade 1 to 2 gastrointestinal toxicity from the start of SBRT to 2 weeks after treatment. There were no grade 3 or greater radiation-related toxicities or delays for cycle 2 of gemcitabine. On endoscopy, there were no grade 2 or higher mucosal toxicities. Two patients had a partial response. The median progression free and overall survival were 6.8 and 12.2 months, respectively. Global QoL did not change between baseline and immediately after radiation treatment.

**Conclusions:**

SBRT with concurrent full dose gemcitabine is safe when administered to patients with LAPC. There is no delay in administration of radiation or chemotherapy, and radiation is completed with minimal toxicity.

## Background

Quality of life (QoL) is of paramount importance when cure is not achievable, as is currently the case with locally advanced pancreatic cancer (LAPC). In the United States, concurrent chemoradiation therapy is commonly considered the standard of care for LAPC patients, but outcomes are still poor. Randomized clinical trials of chemoradiation vs. chemotherapy alone have shown mixed results, with some trials showing a survival benefit with chemoradiation [1-4] and others demonstrating that is has a negative impact on overall survival [5,6]. Furthermore, in trials in which there was a survival advantage, toxicity was often higher in the chemoradiation arm [1,3,4]. Therefore, clinicians and patients must weigh the small potential gain in overall survival versus the potential adverse effects with chemoradiation.

Traditionally, chemoradiation trials include the use of conventional external beam radiation. This technique uses large radiation fields that deliver a high percentage of the prescription dose to surrounding critical structures possibly leading to significant toxicity and decreased quality of life. When irradiating abdominal tumors with conventional external beam radiation, strict adherence to normal structure dose constraints may limit the delivery of the intended radiation dose to the tumor and potentially result in premature local failure and death. Conversely, delivering high doses of radiation to adjacent critical structures without strict dose constraints increases the risk of late radiation induced complications. Intensity-modulated radiation therapy (IMRT) has made advances over 3D conformal treatment in protecting critical structures and reducing toxicity [7,8]; however grade 3 and 4 toxicities are still reported and treatment breaks are required that may reduce the treatment’s effectiveness. Tumor motion due to respiration [9] and unpredictable gastrointestinal distention [10], require large IMRT planning target volume expansions that limit the maximum prescription dose. Furthermore, daily IMRT treatments for approximately six weeks can be taxing to these ill patients and their families and society [11]. SBRT may prevent local progression of disease while sparing nearby critical structures, thus providing an improved durable QoL. Furthermore, SBRT is an appealing option for LAPC since it allows reduced treatment times. Previous studies have investigated SBRT (25 Gy in one fraction) as a boost to IMRT with concurrent 5-FU, and SBRT monotherapy (25 Gy in one fraction) with gemcitabine [12,13]. In both of these trials, local control was excellent; however, late duodenal toxicity was significant, and was related to the amount of duodenum irradiated [14]. Median overall survival was comparable to modern expected outcomes [4,15,16]. In an effort to decrease late duodenal toxicity, we and others [17-20], have examined the use of fractionated SBRT with full dose gemcitabine. Here, we report the results of our feasibility trial.

## Methods

### Patients

Patients with biopsy-proven, non-metastatic, unresectable pancreatic adenocarcinoma who had an adequate performance status and normal hepatic and renal function were eligible for enrollment on this study. Unresectable disease was defined as any greater than 180° encasement of the celiac, superior mesenteric, hepatic, or gastroduodenal arteries, or greater than 2 cm involvement of the portal or splenic veins, or any venous tumor thrombus. Patients with duodenal mucosal involvement (at time of initial endoscopy) were excluded. The Georgetown University institutional review board approved this study and all patients provided informed written consent.

### Treatment summary

**Figure 1:**
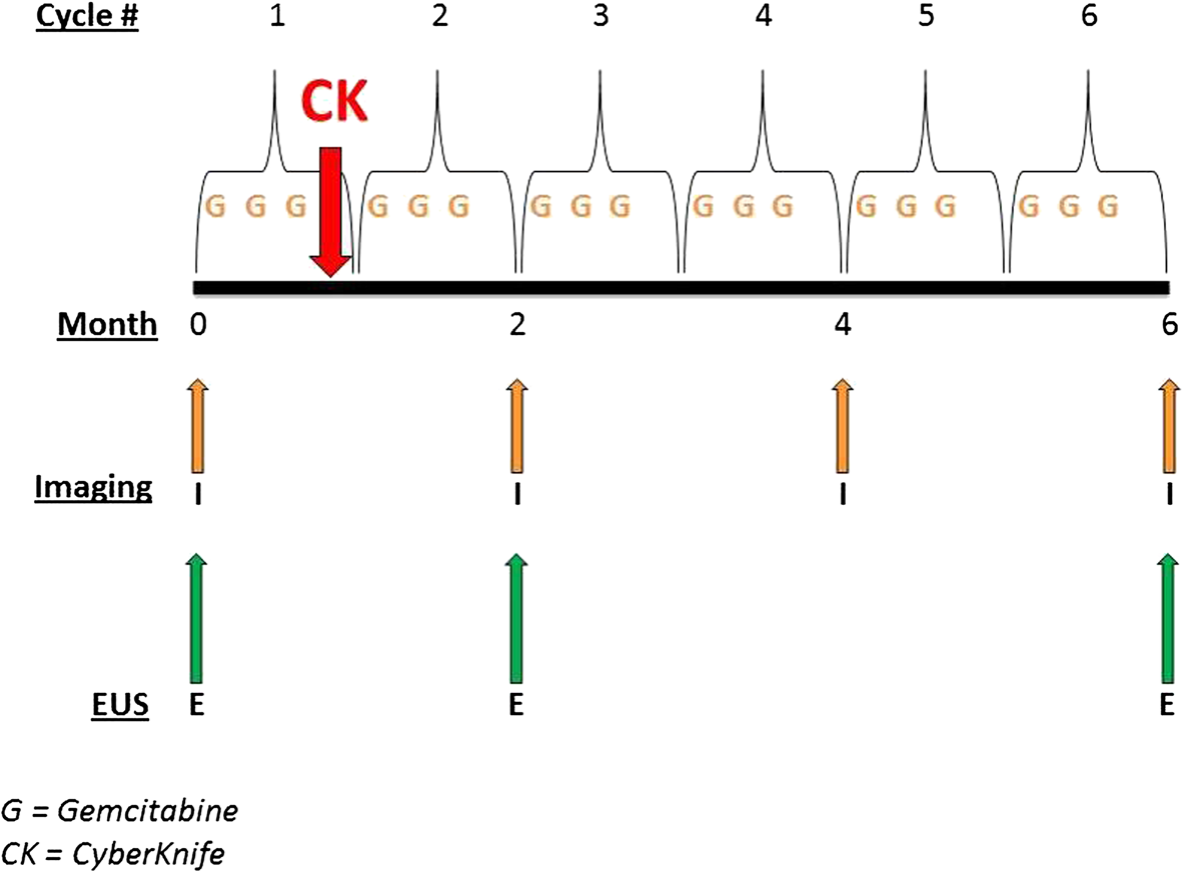
Schema: treatment algorithm and tumor assessment.

Figure 1 provides a schematic overview of patient treatment. Eligible patients first underwent an esophagogastroduodenoscopy with endoscopic ultrasound (EGD/EUS) with placement of gold fiducial markers required for SBRT. Gemcitabine, 1000mg/m^2^, was delivered on day 1 and day 8 (cycle 1, weeks 1 and 2 of gemcitabine). Prior to day 15 (cycle 1, week 3 of gemcitabine), patients underwent a CT scan for SBRT treatment planning. Patients then received day 15 gemcitabine (cycle 1, week 3), and during the subsequent “off” week from chemotherapy (cycle 1, week 4) the patients received SBRT. Cycle 2 of gemcitabine was administered without a planned treatment delay, followed by restaging scans and EGD/EUS to assess tumor response and acute mucosal toxicity. Patients, who were tolerating therapy and without progression as determined by RECIST criteria [21], continued gemcitabine with restaging scans every two cycles, to a maximum of 6 cycles. One additional EGD/EUS was performed after 6 cycles to assess late toxicities of SBRT.

### SBRT

SBRT treatment was delivered with the CyberKnife (Accuray Incorporated, Sunnyvale, CA) which uses real time tumor tracking and requires 3 visible, non-collinear fiducials on the orthogonal x-ray images [22]. Three to five gold fiducials were placed endoscopically in the pancreatic mass as previously described [23]. Seven days after fiducial placement, fine-cut (1.25 mm) treatment planning CT’s with oral and IV contrast were obtained during a full inhalation breath hold.

**Figure 2:**
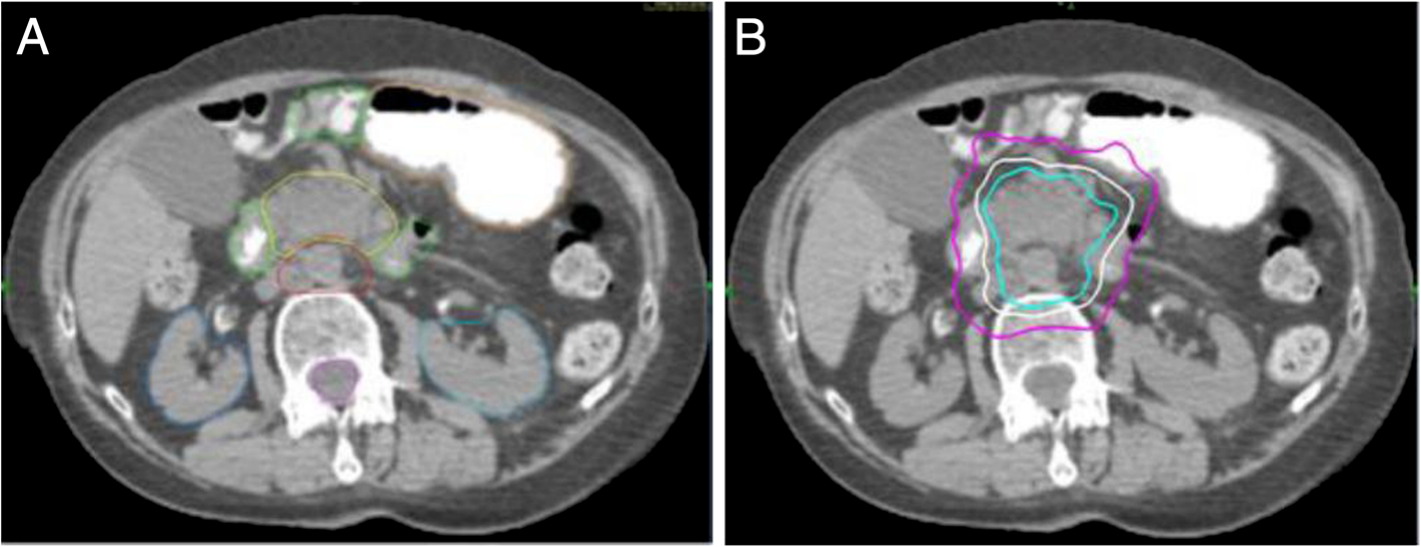
**CyberKnife treatment planning for patient #2: A. Axial computed tomography images demonstrating the gross tumor volume (yellow), adjacent vascular volume (red), duodenum (green), stomach (orange), spinal cord (pink) and kidneys (blue). B**. Isodose lines shown as follows: Blue 81% (prescription), white 70% and purple 50%.

The gross target volume (GTV) included the pancreatic mass (Figure 2). The SBRT planning target volume (PTV) equaled the GTV plus the adjacent vasculature (AV) without expansion. The AV is the area of vasculature involvement [24] and generally included the mesopancreas and posterior nodal regions (posterior pancreaticoduodenal, superior mesenteric, and para-aortic nodes) at the level of the pancreatic mass. Surgical series have demonstrated a high probability of metastases to these regions with 32.31%, 15.85 %, and 10.92%, respectively [25]. Furthermore, the mesopancreas is the primary site for an R1 resection [26]. Therefore, this elective volume was included in increase the probability of an R0 resection if any patients were deemed resectable after chemoradiation. Given the sub-millimeter precision of CyberKnife treatment [22], no uniform expansion was added to correct for set-up inaccuracy. Instead the initial contours of the GTV were generous because computed tomography scans significantly under-represent pancreatic tumor size [27]. SBRT with respiratory tracking was administered as previously described for lung tumors [28]. The prescription dose was 25 Gy delivered to the PTV in 5 fractions of 5 Gy over 5 days. The volume of the PTV receiving 25 Gy, termed the V25 Gy, was to be at least 95%. Variations of the V25 Gy that were less than 95%, but greater than or equal to 90%, were considered minor variations; whereas variations to the V25 Gy that were less than 90% were considered major variations. The prescription isodose line was limited to ≥ 75% which restricted the maximum tumor dose to 133% of the prescription dose. The duodenal and adjacent bowel volumes receiving 25 Gy were limited to < 1 cc. Variations were considered minor if the V25 Gy was ≥ 1 cc and < 2 cc; variations were considered major if the V25 Gy was > 2 cc (Table 1).

**Table 1:** Radiation treatment parameters

**Treatment characteristic**	**Mean**	**Per protocol**	**Minor variations**	**Major variations**
GTV	175 cc			
PTV	360 cc			
Isodose Line	78%	10	0	0
PTV (V25Gy)	95.5%	10	0	0
Other Bowel (V25Gy)	0.40 cc	10	0	0
Duodenum (V25Gy)	0.78 cc	9	1	0

Mean percent target coverage, prescription isodose line and critical structure dose/volume results and minor and major variations from protocol.

### Toxicity assessment

Toxicity was scored according to the National Cancer Institute Common Terminology Criteria for Adverse Events, Version 3.0. Due to the lack of a systematic endoscopic grading system for radiation induced mucosal injury, a previously developed descriptive based scoring system [29] was used to evaluate for extent of telangiectasia, congested mucosa, ulceration, stricture and necrosis.

### Quality of life assessment

All patients were asked to complete two quality of life questionnaires: the EORTC QLQ-C30 version 3 and EORTC QLQ-PAN26, on the first day of each chemotherapy cycle. The QLQ-C30 measures generic health status. QLQ-PAN26 is a pancreatic cancer treatment specific instrument that assesses patient function and bother [30].

### Statistical analysis

Baseline characteristics and adverse events were tabulated. Adverse events were described for each symptom according to Wachter, *et al.*[29] and the NCI CTCAE version 3.0. Each symptom was counted once per patient at the highest grade it occurred. Progression-Free Survival (PFS) was calculated as the date gemcitabine was started to the date of progression or death, whichever came first. Overall survival (OS) was calculated from the start date of gemcitabine to date of death. Since patients were followed until death, no censoring occurred for PFS or OS. OS and PFS were calculated using the Kaplan-Meier method [31]. Median OS and PFS times were reported with 95% confidence intervals (CI). Local control was determined by continued growth of the primary on serial radiologic imaging. Wilcoxon rank sum test was used to assess QoL score changes.

## Results and discussion

### Patient and tumor characteristics

Fourteen patients were screened and three patients were not enrolled for the following reasons: 1) Identification of metastatic disease; 2) Liver transaminitis; and 3) Inability to endoscopically place fiducials due to a previously placed duodenal stent. One patient, who was 81 years of age, withdrew due to significant decrease in performance status after just two radiation treatments so an 11^th^ patient was enrolled for 10 evaluable patients. These patients were treated over a period extending from September 2009 to February 2011 (Table 2) and were followed until death. The mean age at enrollment was 62.5 years (range 50 – 79 years). Nine patients had an ECOG performance status (PS) 1 and one patient was an ECOG PS 0. All the tumors involved the pancreatic head or body and six patients also had clinically involved lymph nodes.

**Table 2:** Patient characteristics

**Patient**	**Age**	**Sex**	**ECOG**	**Location**	**Lymph nodes**
1	62	M	1	Head	N1
2	79	F	1	Head & Body	N1
*3*	Withdrew before completing radiation				
4	Screening failure due to metastatic disease				
5	74	M	1	Body	N1
6	50	M	1	Body	N0
7	63	F	0	Head	N1
8	56	F	1	Head	N1
9	63	M	1	Head	N0
10	76	M	1	Head	N1
11	Screening failure due to elevated liver function.				
12	Screening failure due to inability to place fiducials.				
13	57	F	1	Head	N0
14	62	F	1	Head	N0

### SBRT

All patients completed SBRT as prescribed. The mean PTV was large with a mean volume of 360 cc (range, 154 cc – 548 cc) and the mean GTV was 175 cc (range, 103 – 336 cc) (Table 1). Radiation was delivered to a mean prescription isodose line of 78% (range, 75% - 83%) in 5 treatments (Table 1). The mean percent target coverage was 95.42%. Treatment plans were conformal with a mean new conformity index (the target volume multiplied by the prescription isodose volume divided by the target volume covered by prescription isodose volume squared [32]) of 1.39. Plans were inhomogeneous by design (mean homogeneity index of 1.28) to minimize dose to adjacent critical structures. There were no major protocol variations; one minor protocol variation occurred when 1.0 cc of duodenum received 25 Gy (Table 1).

From the start of SBRT and for 1–2 weeks thereafter, patients generally exhibited grade 1–2 nausea and abdominal cramping, but there were no grade 3 radiation-related acute toxicities, and cycle 2 of gemcitabine was not delayed. Serial endoscopy showed 60% of patients had asymptomatic, small mucosal ulcerations (Grade 1) at 2 months. Of the five patients who completed endoscopy at six months one patient had residual grade one mucosal congestion. There were no grade 2 or higher mucosal toxicities.

### Chemotherapy

Overall, 8 of 10 patients completed 6 cycles of gemcitabine, although all patients required dose reductions or schedule modifications, mostly due to myelosuppression from the chemotherapy. Of the two patients who did not complete all six cycles, one patient became critically ill during cycle three, was admitted for sepsis and taken off study. The other patient progressed with distant metastases after chemotherapy cycle five and was changed to an alternative chemotherapy regimen.

### Patient outcomes

**Figure 3:**
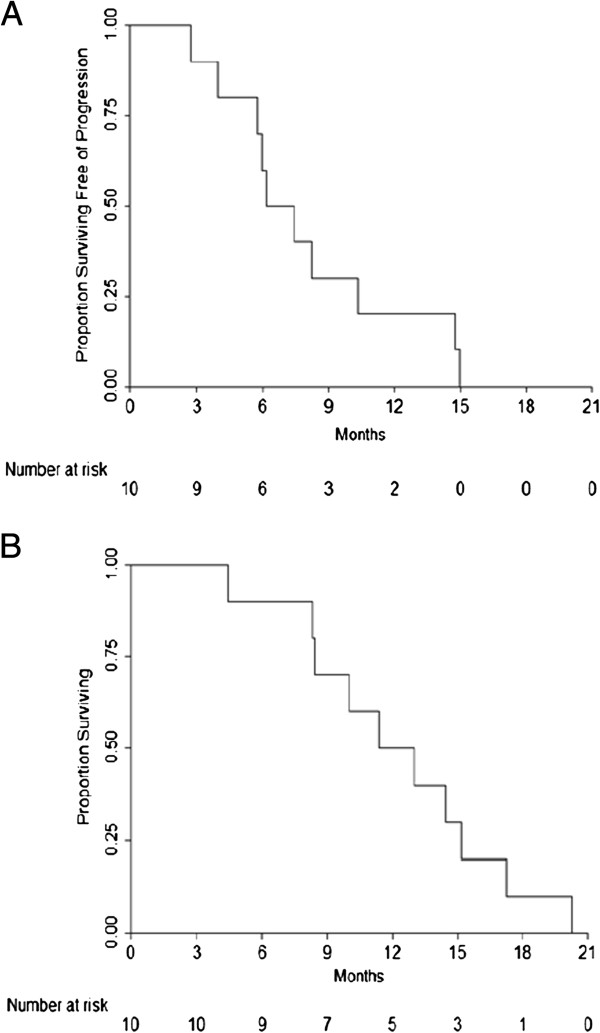
Kaplan-Meier plot of (A) progression-free survival (PFS) and (B) overall survival (OS).

All patients were followed until death. Two patients had a partial response by RECIST. None of the patients were rendered resectable. All but one patient experienced disease progression by RECIST with a median time to progression of 6.8 months (95% CI: 2.8, 10.3 months). Four patients experienced local progression as the first site of failure and a total of six patients experienced local progression before death (Table 3). The median survival was 12.2 months (95% CI: 4.4, 15.2 months) (Figure 3). Of the patients who completed all cycles, one patient received maintenance chemotherapy with capecitabine and three patients received salvage chemotherapy and/or conventional radiation therapy (39 cGy in 1.8 Gy fractions) at the time of progression. Patients who did not receive salvage therapy either opted not to or were too sick to do so (Table 3).

**Table 3:** Individual clinical outcomes

**Patient**	**Best response (RECIST)**	**Time to progression (days)**	**First site of progression**	**Salvage therapy**	**Time to death (days)**
1	SD	189	Local + distant	None	254
2	PR	317	Local	Capecitabine (maintenance); Radiation	440
5	SD	470	Distant	None	490
6	SD	228	Distant	FOLFOX	397
7	SD	176	Distant	Erlotinib with gemcitabine	382
8	PR	450	Local	None	462
9	SD	251	Distant	Capecitabine, FOLFOX+ABT888	618
10	PD	66	Local	None	135
13	SD	168	Distant	None	257
14	SD	121	Distant	Progressed on gemcitabine, switched to lapatinib + capecitabine	305

### Quality of life

**Figure 4:**
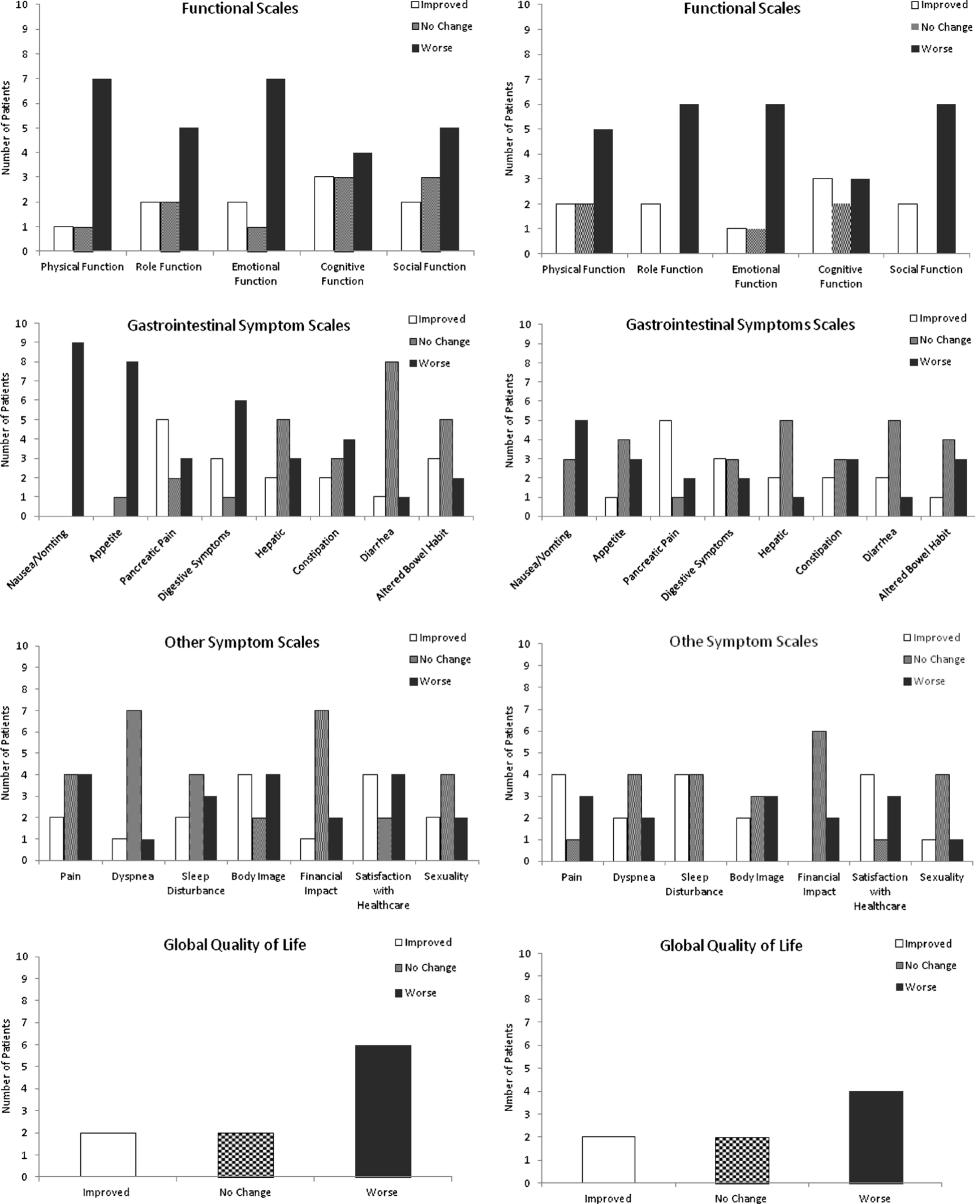
**Quality of life.** Number of patients who had improvement, no change, or worsening between baseline and immediately after SBRT and from baseline to one month after SBRT. Numbes may not add to 10 due to incomplete questionnaires.

Of the eight patients who received all six chemotherapy cycles, only four patients completed both the EORTC QTC 30 and PAN 26 questionnaires for all chemotherapy cycles, which limited the analysis to the first three chemotherapy cycles. QoL scores immediately after SBRT showed a statistically significant increase from baseline for fatigue, nausea/vomiting (N/V) and anorexia (p < 0.05). The declines in fatigue and anorexia QoL were no longer statistically different from baseline by chemotherapy cycle 3. Symptoms did not improve significantly after radiation therapy; however, there was a trend towards improved back pain, night pain and abdominal discomfort. Global QoL did not change significantly from baseline due to radiation treatment (Figure 4).

In pancreatic cancer, improved methods of controlling the primary cancer are needed as local progression adversely affects the length and quality of life. Dose escalation with standard external beam radiation therapy has been attempted [33]. However, the inability to limit normal tissue doses led to severe life-threatening toxicity. On the other hand, standard radiation treatment for 5–6 weeks prolongs treatment time and delays administration of systemic therapy. Recent data suggest that large radiation fraction sizes are radio-biologically favorable over lower fraction sizes in slow growing tumors such as pancreatic cancer [34,35]. This is supported by trials that achieved improved pancreatic cancer local control rates by utilizing large fraction sizes with intraoperative radiation therapy or brachytherapy [36-42]. Analysis of these data suggested that radiobiologic dose escalation to the tumor volume could improve local control in pancreatic cancer.

Gemcitabine is a potent pancreatic cancer radiosensitizer [43]. As previously shown, by the Eastern Cooperative Oncology Group, in patients with LAPC, concurrent gemcitabine at a reduced dose (600 mg/m^2^) and radiation (50.4 Gy in 28 fractions) provided survival benefit over gemcitabine (1000 mg/m^2^) alone (4). Furthermore, full dose gemcitabine (1000 mg/m^2^/week) with concurrent radiation therapy has been shown to be feasible and safe with the omission of elective nodal irradiation and radiation dose reductions [15]. The combination of full dose gemcitabine with radiation therapy should enhance local and systemic tumor control.

In our study, patients received full dose gemcitabine (1000 mg/m^2^) and concurrent SBRT with an included elective nodal volume The radiation dose given was 25 Gy in five consecutive fractions, which is an equivalent dose in 2 Gy fractions of approximately 40 Gy. We felt that this would be a safe but effective dose drawing from the University of Michigan’s IMRT dose escalation trial. In their trial the MTD was determined to be 36 Gray given in 2.4 Gray fractions, which is equivalent to 41.4 Gray in 1.8 Gray fractions [44]. Furthermore, the inhomogenity is much greater with SBRT than IMRT and consequently the central tumor dose is significantly higher than the prescription dose to the periphery.

Hypofractionated stereotactic body radiation therapy was completed in one week in all patients. Critical organs including the bowel, liver, spinal cord, and kidneys were spared. There were no grade 3 or 4 acute toxicities secondary to radiation and the initiation of the second cycle of chemotherapy was not delayed. Our protocol had strict duodenal dose restrictions and additionally monitoring for late toxicity by endoscopy was performed. With our dose limitations no late toxicity was observed. Chemotherapy toxicity from full dose gemcitabine was as expected with the majority secondary to adverse hematologic effects. However, our local control was lower than that achieved with a single fraction of 25 Gy. This is despite the fact that our patients received concurrent full dose (1000 mg/m^2^) gemcitabine rather than sequential chemotherapy. This may be due to the relatively low biological effective dose used in this trial.

Despite the lower local control, our median progression free survival and overall survival of 6.8 and 12.2 months are comparable to contemporary chemoradiation trials. Quality of life scores were difficult to interpret due to the small number of patients that completed all the questionnaires. This could have biased the results since patients who progressed were taken off study and did not complete the questionnaires and patients too ill to complete the questionnaires would most likely report lower QOL scores.

## Conclusions

Our results from this pilot study demonstrate the feasibility and tolerability of delivering SBRT with concurrent full dose gemcitabine. Unfortunately, local and distant progression remains the predominant patterns of failure for these patients. Nonetheless, SBRT remains a useful tool to optimize available treatment for patients with tumors in close proximity to critical structures while maintaining QOL. Additional studies combining SBRT with regimens that have been proven to improve the control of systemic disease, such as FOLFIRINOX, are also being evaluated [45].

We are planning to conduct a Phase II trial to confirm the efficacy of this treatment. While outcomes were comparable to contemporary trials, this study only included ten patients. Furthermore, the SBRT dose used in this trial is currently lower than what our institution is safely using at this time. With the low toxicity profile experienced by patients on this trial we will increase the SBRT dose and our future trial will most likely treat patients in five consecutive fractions of 6 Gy per fraction. This will hopefully provide improved local control. While there is still an issue with controlling distant disease, local tumor progression leads to significant morbidity.

## Abbreviations

QoL: Quality of life; LAPC: Locally advances pancreatic cancer; SBRT: Stereotactice body radiation therapy; IMRT: Intensity-modulated radiation therapy; EGD: Esophagogastroduodenoscopy; EUS: Endoscopic ultrasound; GTV: Gross target volume; PTV: Planning target volume; AV: Adjacent vasculature; PFS: Progression-free survival; OS: Overall survival.

## Competing interests

Sean P. Collins is an Accuray clinical consultant.

## Authors’ contributions

MKG assisted with data collection and writing of the manuscript. SPC help conceive the study and design it. In addition he managed patients on protocol and contributed to the writing of the manuscript and editing. RS served as the statistician for the study. GT assisted in data collection and analysis for the quality of life component. AC carried out the esophagogastroduodenoscopy/endoscopic ultrasound on patients. LL was the nurse coordinator for the study and assisted with data collection. LB helped with data coordination and collection. SL was the dosimetrist and carried out radiation treatment planning. SS assisted with data collection. NH AC carried out the esophagogastroduodenoscopy/endoscopic ultrasound on patients. RJ provided the radiologic review on all patients. CDJ assisted with the quality of life analysis and manuscript review. PJ provide patient management and assisted in patient selection. JLM assisted with study design and patient management. MJP conceived the study and contributed to its design. He managed patients on study and contributed to the writing of the manuscript and editing. All authors reviewed and approved the final manuscript.

## Funding

This trial was funded by the Otto J. Ruesch Center for the Cure of Gastrointestinal Cancers and supported by NIH Grant P30CA051008.
